# Vagal Activity and Fat Oxidation Basal Correlates in Older Active Postmenopausal Women: A Cross-Sectional Study

**DOI:** 10.1186/s40798-026-01004-1

**Published:** 2026-03-12

**Authors:** Jordi Monferrer-Marín, Ainoa Roldán, Jørn Wulff Helge, Cristina Blasco-Lafarga

**Affiliations:** 1 Sport Performance and Physical Fitness Research Group (UIRFIDE), Physical Education and Sports Department, University of Valencia, Valencia, Spain; 2 Department of Biomedical Sciences, Faculty of Health and Medical Sciences, University of Copenhagen, Copenhagen, Denmark

**Keywords:** Autonomic nervous system, Baroreflex, Energy expenditure, Heart rate variability, Metabolic flexibility, Total power, Vagus nerve

## Abstract

**Background:**

Heart Rate Variability at rest has been recently associated with metabolic outcomes alongside exercise, which in turn have been associated with energy expenditure, muscle power and fat mass. This study aimed to analyse the relationship at rest between autonomic function and metabolic outcomes, in physically active postmenopausal women. We hypothesised that, autonomic function is more strongly associated with Fat oxidation than basal metabolic rate. In sixty-one active postmenopausal women (67.9 ± 5.3 years; 40.3 ± 4.3 kg muscle mass) basal metabolic rate and Heart Rate Variability analysis was recorded simultaneously for 30 min with participants resting supine under standardised activity and diet conditions.

**Results:**

Root Mean Square of Successive Differences of heart beats (RMSSD) and Total power showed a predictive value for resting Fat oxidation (β = 0.46; β = 0.26), explaining 30% of the variance. Including traditional predictors such as energy expenditure increased explained variance to 57.5%. In this model, RMSSD association disappeared, Total power (β = 0.88) became the strongest predictor, and together with energy expenditure (β = 0.53), showed significant associations with FATox. Respiratory exchange ratio only correlated with RMSSD (β = − 0.54) in the isolated Heart Rate Variability model, without basal metabolic rate associations. Box plots of RMSSD quartiles revealed a difference in fat-oxidation between the highest and lowest quartiles, a pattern not seen for Total power.

**Conclusion:**

Baroreflex activity and fat oxidation associate at rest in active postmenopausal women with preserved cardiovascular function. Total power emerges as the strongest Heart Rate Variability predictor of baseline Fat oxidation in the multivariable models. Stratification by RMSSD quartiles revealed graded differences in fat oxidation rates across levels of vagal modulation.

**Trial registration:**

Clinical Trials, NCT06336070. https://clinicaltrials.gov/study/NCT06336070 Registered: 4 April 2024.

**Supplementary Information:**

The online version contains supplementary material available at 10.1186/s40798-026-01004-1.

## Key Points

- Data reveal a robust association between cardiac vagal activity, measured through RMSSD and Total power, and the rate of Fat oxidation at rest in physically active postmenopausal women. Non-linear variables such as detrended fluctuation analysis (DFA) do not associate under basal conditions.
- Despite the age-related decline, the postmenopausal active women displayed preserved autonomic and metabolic responses, evidenced by moderate sympathetic index and maintained RMSSD values, low respiratory exchange ratios and high Fat oxidation rates.
- A combined predictive model further establishes that Total power, indicative of baroreflex sensitivity, more accurately predicts resting Fat oxidation than overall energy expenditure, suggesting that HRV signals may be key markers of metabolic health in this aging population.

**Supplementary Information:**

The online version contains supplementary material available at 10.1186/s40798-026-01004-1.

## Background

The rising incidence of cardiovascular disease has led to a growing interest in the study of cardiac activity and autonomic nervous system (ANS) function in the later decades. Recent studies have identified the vagus nerve as a key player in regulating blood flow to the heart. This helps to maintain adequate cardiac output without causing oxygen shortages [1]. Conversely, ageing is associated with a vagus nerve dysautonomia [2], which results in an increase in basal sympathetic activity and a decrease in both sympathetic and parasympathetic reactivity [3]. This autonomic dysfunction has repercussions that extend beyond cardiovagal control [4], affecting also inflammatory processes [5], motor function [6], and even energy metabolism.

Specifically, in metabolism the vagus nerve plays a key role, having direct effects on adipose tissue by activating β-adrenergic receptors, thereby enhancing thermogenesis and promoting lipolysis within adipose depots [7]. At the hepatic level, its action dynamically modulates glucose release or storage according to physiological demands [8]. Hence, greater efficiency of the autonomic nervous system has been associated with higher endogenous lipid oxidation [9], suggesting a possible influence of autonomic function on fat oxidation, which could be affected by ageing.

In this context, heart rate variability (HRV) serves as a non-invasive marker of ANS function and cardiovascular health by examining reflex signals originating in the brain, mediated by the sympathetic and vagus nerves that innervate the sinoatrial node [10]. HRV is also a significant clinical marker for predicting cardiovascular risk and overall mortality [11]. Recent evidence in postmenopausal women has revealed a significant association between resting HRV non-linear indicators and a higher rate of maximal fat oxidation (MFO) measured by an incremental exercise test [12]. This finding could indicate autonomic function as a non-controllable factor influencing metabolic flexibility in exercise, together with other variables with strong influence such as body composition, age and training status [13]. Nonetheless, the topic is relatively new and presents many gaps in knowledge, such as the possible influence of autonomic functioning in substrate oxidation rates under resting conditions, and the possibility of analysing this influence through HRV variables.

In this scenario, research regarding resting metabolism and autonomic responses has been, to date, restricted to basal energy expenditure assessment, which has demonstrated an inverse correlation with vagal activity in a cohort of young women [14]. This suggests a mediating role of the sympathetic nervous system in the interaction, contributing to the increase in basal energy expenditure [15]. However, with ageing this sympathetic activation may be attenuated [16], due to the influence of other factors such as a progressive reduction in muscle mass [17] and endocrine changes as oesteogenic decline in women after menopause [18]. It’s crucial to consider how body composition [19], training status, muscle power [20], and energy expenditure [21] influence the rate of fat oxidation at rest in the older population. Understanding these factors is key to elucidate the interplay between HRV and fat oxidation, which is the purpose of this study.

Therefore, the present study aim to analyse the association between baseline autonomic function and fat oxidation at rest in a sample of physically active postmenopausal women. Specifically, the objective is to examine the potential relationship of vagal activity (i.e. HRV variables) with fat oxidation at rest, respiratory exchange ratio (RER), and basal metabolic rate (BMR), thereby identifying the key variables underlying this interaction between both sides of the cardiovascular health (metabolic and autonomic function). The hypothesis is that the rate of fat oxidation is more closely related to autonomic function than the BMR, with a degree of association similar to that observed for muscle power or fat mass, variables traditionally associated with fat oxidation.

## Methods

### Study Design

This is a cross-sectional study based on the recording of basal metabolic rate at rest in postmenopausal women from the POWER Health Study registered as a clinical trial (NCT06336070). All participants volunteered to participate in the study and signed written informed consent and was performed in accordance with the standards of ethics outlined in the Declaration of Helsinki (2024-FIS-3251696).

### Participants

61 active postmenopausal women (67.9 ± 5.3 years; 40.3 ± 4.3 kg muscle mass) completed the two-day study with at least 48 h rest between each protocol. The inclusion criteria were as follows: (1) women over 55 years, (2) moderately active according to the International Physical Activity Questionnaire (IPAQ), and (3) no medical contraindications for physical exercise. The exclusion criteria included: (1) no chronic disease, (2) no use of medications (e.g., beta-blockers) that limit or affect physical activity, (3) not undergoing hormone replacement therapy or any estrogen treatment, and 4) no episodes of hypotensive response to exercise. All participants volunteered to participate in the study and signed written informed consent after receiving complete information regarding the aim of the study and potential side effects of the procedures.

### Main Procedures

On the first day, baseline measurements were taken, including blood pressure with Omron M6 sphygmomanometer (Omron Healthcare Co., Ltd., Muko, Japan), oxygen saturation assessed using Nonin Onyx Vantage 9590 (Nonin Medical, Inc., Plymouth, United States), and heart rate variability (HRV) at rest for 10 min recorded by Polar H10 chest band device (Polar Electro Oy, Kempele, Finland). Anthropometric measurements were obtained using a portable stadiometer (Seca model 222, Seca, Hamburg, Germany), while body composition was assessed through bioimpedance (Tanita DC-430 MA S; Tokyo, Japan). Muscle power was evaluated using the five sit-to-stand test (5STS), and capillary blood samples were collected from the index finger of the left hand to measure blood lactate levels using the Lactate Scout Sport Solo device (SensLab GmbH, Berleben, Germany). The device has demonstrated acceptable validity and precision for field and laboratory measurements in comparison with reference analyzers [22], and it was calibrated according to the manufacturer’s instructions before each measurement session. Nutrition was standardised from the evening meal prior to the day of recording, ensuring a total energy intake of over 900 kJ at dinner, with at least 550 kJ coming from carbohydrates. Participants attended the assessment after a 12 h overnight fast, following the recommendations of Fullmer et al. [23].

On the second day, participants arrived well-rested in the early morning (8:30 a.m. or 10:00 a.m.), having avoided vigorous physical activity for the previous 48 h and moderate physical activity for the last 24 h. After following the same nutritional control, participants were assessed in the same laboratory using identical equipment, protocols, and evaluators. Following a 15-min seated rest, a 30-min basal metabolic rate (BMR) was conducted. This analysis was performed using a Cosmed K5 metabolic cart (Cosmed, Rome, Italy), while HRV was monitored simultaneously, in the supine position in a quiet room with controlled environmental conditions (22–24 °C), dim lighting, with controlled noise exposure and personnel presence, minimizing environmental influences on autonomic regulation and substrate oxidation.

### Basal Metabolic Rate Analysis

The 30-min BMR recording by Cosmed K5 (Cosmed, Rome, Italy) was exported to an Excel sheet for analysis. The first 5 min were discarded, given the transient variations in oxygen consumption and CO₂ production in this period that may alter the accuracy of the calculation [23]. From the remaining 25-min window, steady-state conditions were verified (coefficient of variation ≤ 10% for VO₂ and VCO₂), and mean values across the entire 25-min window were used for all subsequent calculations, BMR (in kilocalories per day [kcal/day]) was estimated using the equation proposed by [24], along with carbohydrate and fat oxidation rates (CHOox and FATox, respectively) using the Frayn Equations [25], assuming no urinary nitrogen excretion. Mean RER, VO_2_, energy expenditure, fat contribution to this energy expenditure (%Fat), respiratory frequency (Rf) and tidal volume (VT) were calculated by using the Cosmed K5 metabolic card software in 25-min windows, which allows this and other variables to be obtained in real time. For subsequent analyses, fat oxidation was expressed relative to muscle mass (RelFATox), obtained from bioimpedance (Tanita DC-430 MA S; Tokyo, Japan) which was always carried out by the same evaluator, in line with previous studies reporting the relevance of body mass and body composition–adjusted expressions of fat oxidation for physiological interpretation [26].

### Heart Rate Variability Analysis

RR interval recordings during the 30-min BMR using the Polar H10 chest strap device were exported from the Polar Sensor Logger application to Kubios Scientific software (version 4.0.2; Biosignal Analysis and Medical Imaging Group, Department of Physics, University of Kuopio, Kuopio, Finland) for further analysis, using the same 25-min analysis window as the metabolic analysis, with the first 5 min excluded to guarantee the time between successive R waves (RR) stability. Artifacts were identified and corrected using the Kubios “automatic method” with a Lambda value of 500 [27], and recordings exceeding 2% of artifacts were excluded (n = 5) from the analysis [28]. The first 5-min were again discarded [23], to ensure consistency in comparing capacities. Time domain, frequency domain, and non-linear dynamics variables were considered.

In the time domain, the root mean square root of successive normal beat-to-beat differences (RMSSD, in milliseconds) was selected as the primary linear variable, reflecting vagal functioning [29]. The PNS index was used to assess parasympathetic cardiac activity, which increases with higher respiratory sinus arrhythmia [30]. The SNS index, which serves as the counterpart to the PNS index, was also included to evaluate cardiac sympathetic activity[31].

For the frequency domain analysis, low-frequency bands (LF, 0.04–0.15 Hz), reflecting baroreflex activity, and high-frequency bands (HF, 0.15–0.4 Hz), influenced by respiratory sinus arrhythmia, were selected as indicator of parasympathetic activity [10]. These measures were complemented by Total power, representing the signal energy of all frequency bands combined, which describes the overall autonomic regulatory capacity of the nervous system [10].

In terms of non-linear dynamics, one variable was evaluated for each of the geometric, entropy, and fractal methods. The geometric approach involved analyzing the standard deviation of the Poincaré plot perpendicular to the line of identity (SD1), which serves as a marker of baroreflex sensitivity [32]. Entropy analysis was conducted using Sample Entropy (SampEn), which measure the complexity of time series data and provides valuable information on cardiovascular functionality and sympathovagal balance [33]. For fractal analysis, the Detrended Fluctuation Analysis (DFA) algorithm was applied to explore correlations between RR intervals across multiple time scales [34]. This method assesses the adaptability and flexibility of the cardiac system, with lower self-similarity indicating a more randomly structured and adaptive system [35]. Specifically, short-term or alpha 1 correlation derived from DFA (DFAalpha1) reflect vagal activity [10]. The window size for DFAalpha1 analysis was set to span 4 to 16 beats [36]. These HRV metrics capture the beat-to-beat activity of the autonomic nervous system, reflecting homeostatic adjustments in cardiovascular control. Such adjustments arise from multisystem networks comprising sensory afferents, integration within brainstem centers and efferent autonomic pathways [37].

### Statistical Analysis

Statistical analyses and figures were performed using RStudio 4.0.2 (R Core Team, Vienna, Austria). After testing for normality (Shapiro–Wilk test), descriptive statistics, including mean, 95% confidence interval and coefficient of variation (CV%), used to assess the relative dispersion and variability of the measured parameters, were calculated for sample characteristics and for descriptives of the primary endpoints of resting Fat oxidation and heart rate variability. A pearson correlation was performed between the main gas exchange predictor variables (RelFATox, BMR and RER), to study their relationship, a multiple linear regression analysis was applied to study the predictive value of HRV on these three metabolic variables. To ensure the viability of using RelFATox as a metabolic variable, sensitivity analyses examined the consistency of associations between absolute, body-mass normalised, and fat-free-mass–normalised fat oxidation using age-adjusted linear regression models both autonomic (RMSSD) and metabolic variables (EE). A full multivariable linear regression model including all candidate autonomic variables was first fitted. Model refinement was then performed using a backward elimination strategy guided by multicollinearity diagnostics (variance inflation factors) and model parsimony. The first regression models were run to determine the HRV variables to be included in the model, based on multicollinearity, which excluded LF power, HF power, PNS index and SD1. Other variables such as SampEn and the SNS index were eliminated from further analysis, due to no predictive capacity and a concomitant attenuation of the predictive value of the determinant HRV variables (see supplementary material). RMSSD, Total Power and DFAalpha1, three HRV variables from different HRV analysis domains formed the final model, in order to determine the predictive value on the main metabolic variables.

In order to ascertain the primary predictor variables of RelFATox at rest, and to determine the significance of autonomic function in the combined model, the two principal HRV variables (RMSSD and Total Power) were selected along with other variables traditionally associated with the rate of Fat oxidation, such as energy expenditure, age, fat mass and muscle power in the 5STS test.

Finally, a one-way ANOVA test was conducted testing for differences in RelFATox and RER values between the RMSSD quartiles, together with their box plots.

## Results

### Study Population

Sixty-one participants over 55 years were analysed. Their descriptive characteristics are reported in Table 1. Data showed a maintained relative power of 5STS test, as reported in similar studies [38]. Basal blood lactate remained low, although with a high dispersion.

**Table 1:** Baseline characteristics of the study population (n = 61)

Variable	Mean	95% CI lower bound	95% CI upper bound	CV%
Age (yrs)	67.8	66.4	69.2	7.8
Weight (kg)	66.0	63.1	69.0	17.3
Fat Mass (%body mass)	34.7	33.1	36.3	16.6
Fat Mass (kg)	23.5	21.5	25.5	33.2
MM (kg)	40.4	39.2	41.5	11.0
SBP (mmHg)	127	123	131	11.6
DBP (mmHg)	77	75	79	10.4
HR (bpm)	62.4	63.5	67.4	11.8
Relative 5STS Power (W/kg)	3.3	3.1	3.4	17.6
Baseline lactate (mmol/l)	1.4	1.2	1.6	47.2

*95% CI*, 95% confidence interval; *CV%*, coefficient of variation; *L-MFO*, low MFO group; *H-MFO*, high MFO group; *yrs*, years; *MM*, muscle mass; *SBP*, systolic blood pressure; *DBP*, diastolic blood pressure; *HR*, heart rate; *5STS*, five times sit-to-stand test

As shown in Table 2, BMR was found to be below 1000 kcal/day, with a RER of less than 0.80, both with a lower dispersion compared with others metabolic variables (27.37% BMR and 6.87% RER) since fat oxidation displayed greater dispersion (≈50% CV in both absolute and relative fat oxidation) [14]. Ventilatory parameters were consistent with resting conditions, with a low respiratory frequency and modest tidal volume, however, both variables exhibited moderate-to-high inter-individual variability, reflecting heterogeneous ventilatory patterns at rest.

**Table 2:** Cardiovascular data of baseline metabolic rate measurements along 25 min

Variable	Mean	95% CI lower bound	95% CI upper bound	CV%
*Gas exchange parameters (n* = *61)*				
VO2 (ml/min/kg body mass)	3.12	2.93	3.30	24.6
RER	0.79	0.78	0.81	6.9
%Fat	69.7	65.6	73.8	23.3
Fat oxidation (g/min)	0.07	0.06	0.08	48.5
Relative Fat oxidation (mg/min/MMkg)	1.85	1.63	2.06	46.2
BMR (kcal/day)	965	898	1032	27.4
BMR (kcal/day/kg)	14.8	13.9	15.7	24.5
Energy expenditure (kcal/min)	0.96	0.89	1.02	27.4
Rf (breaths/min)	13.71	12.92	14.49	22.61
VT (L)	0.50	0.46	0.55	34.23
*HRV parameters (n* = *61)*				
SNS index	0.98	0.63	1.32	137
RMSSD (ms)	29.1	21.2	37.0	105
VLF power (ms^2^)	68.1	56.9	79.25	64.9
LF power (ms^2^)	402	304	500	96.1
HF power (ms^2^)	350	216	483	151
Total power (ms^2^)	745	537	953	107
SD1 (ms)	20.7	15.0	26.3	105
SampEn	1.72	1.65	1.78	14.6
DFAalpha1	0.93	0.87	0.99	24.9

Data are presented as mean ± standard deviation*.* *VO2*, oxygen consumption; *RER*, respiratory exchange ratio; *%Fat*, percentage of energy from fat durning the test; BMR, basal metabolic rate; *MMkg*, muscle mass in kilograms; *Rf*, respiratory frequency; *VT*, tidal volume; *HRV*, heart rate variability; *SNS index*, sympathetic index; *RMSSD*, root mean square of successive beats deviation; *LF power*, low frequency band (0.04–0.15 Hz) in the power spectral density; *HF power*, high frequency band (0.15–0.40 Hz) in the power spectral density; *Total power*, the sum of power across all frequency bands in the power spectral density; *SD1*, ellipse’s width of Poincaré Plot; *SampEn*, sample entropy; *DFAalpha1*; detrended fluctuation analysis of short term

HRV analysis pointed out a parallel drop-in parasympathetic activity reflected by low RMSSD and a low Total power, along with higher SNS index as marker of sympathetic activity [14]. The HRV parameters showed a higher dispersion with values higher than 100% in the RMSSD, Total power or the SNS index. In contrast, despite the variability in ventilatory patterns, non-linear HRV indices (SampEn and DFA) showed greater homogeneity, suggesting a more stable organization of cardiac autonomic dynamics under basal metabolic conditions.

The main resting gas exchange parameters BMR and RER was not significantly correlated despite both gas parameter showed a moderate significant association with relative Fat oxidation rate (BMR: r = 0.55, *p* < 0.05; RER: r = − 0.71, *p* < 0.05, respectively).

Sensitivity analyses using alternative fat oxidation scaling approaches showed consistent associations for RMSSD (autonomic marker) and EE (metabolic marker). RMSSD showed stable positive associations with fat oxidation regardless of whether it was expressed in absolute terms or normalized to fat-free mass or muscle mass (β = 0.001; β = 0.014; β = 0.015). Similarly, EE was positively associated with fat oxidation across all scaling approaches (β = 0.088; β = 1.697; β = 1.787).

Regarding the HRV as predictors of Fat oxidation rate during basal conditions (Table 3), RMSSD showed a greater predictor capacity (β = 0.46) compared to Total power and DFAalpha1, explaining 30% of the variance. Total power also had predictive value (β = 0.26); but the variance was reduced to 21%, while DFAalpha1 did not show RelFATox predictability. Despite RMSSD and Total power together were able to explain 35.4% of the variability in FATox, no significant individual influences between HRV variables with each other (*p* = 0.75) were observed in the regression model, nor between RMSSD and Total power (*p* = 0.56), or RMSSD and DFAalpha1 (*p* = 0.37), or between Total power and DFAalpha1 (*p* = 0.76).

**Table 3:** HRV—factors predicting Fat oxidation rate during basal metabolic rate test

Predictor	Estimate	SE	T	*P*	Standardized Estimate (β)	R^2^
Intercept	1.11	0.58	1.91	0.06		
RMSSD	0.01	< 0.01	2.93	0.01	0.46	0.30
Total power	< 0.01	< 0.01	2.08	0.04	0.26	0.21
DFAalpha1	0.18	0.53	0.33	0.74	0.05	0.10

*SE*, Standard Error; *RMSSD*, root mean square of successive beats in miliseconds; *Total power*, the sum of power across all frequency bands in the power spectral density

As mentioned in the methodology section, other variables such as SampEn and SNS index had no predictive effect, thereby diminishing the significance of Total power in the model. HF & LF power, as well as SD1 and PNS index were removed from the model due to multicollinearity problems.

Other potentially influential variables such as respiratory rate was included in these models and were shown to be independent of the fat oxidation rate, as well as influencing the predictive capacity of RMSSD and Total Power, given the multicollinearity between this respiratory variable and HRV, as described in the supplementary material.

Noteworthy, RMSSD was also a predictor of RER (β = − 0.54) under baseline conditions, explaining 14% of the variance. In contrast, neither DFAalpha1 nor Total power were associated to RER.

Regarding BMR, multiple linear regression analysis showed no significant association with the variables RMSSD, Total power and DFAalpha1 (F = 1.20 (3,56), *p* = 0.32).

Following the identification of the HRV variables that exerted the greatest influence on the rate of Fat oxidation (RMSSD & Total power), an additional regression model was constructed with variables traditionally associated with FATox. The inclusion of age, energy expenditure, muscle power and/or fat mass (Table 4), should allow a more precise delineation of the degree of HRV-independent influence. Analysis of variance revealed that this model was statistically significant (F (6, 36) = 8.116; *p* < 0.001). The model explained 57.5% of the total observed variability, which was reduced to 50.4% after adjustment for the number of predictors included.

**Table 4:** Factors predicting fat oxidation rate during basal metabolic rate test

Predictor	Estimate	SE	T	*p*	Standardized Estimate (β)	R^2^
Intercept	0.78	1.86	0.42	0.68		
RMSSD	− 0.02	0.02	− 1.11	0.28	0.49	0.27
Total power	< 0.01	0.00	2.04	0.04	0.88	0.28
Fat Mass	< − 0.01	0.02	− 0.09	0.93	− 0.01	0.03
Relative 5STS Power	− 0.27	0.20	− 1.37	0.18	− 0.17	0.08
Energy Expenditure	1.90	0.43	4.44	< 0.001	0.53	0.36
Age	< 0.01	0.02	0.13	0.89	0.02	0.00

*SE*, Standard Error; *R*^2^, individual coefficient of determination; *RMSSD*, root mean square of successive beats in milliseconds; *Total power*, the sum of power across all frequency bands in the power spectral density; *Relative 5STS Power*, power relative to body mass in five sit-to-stand test

As reflected in Table 4, the primary predictor variable of Fat oxidation rate at rest was the autonomic variable Total power (β = 0.88), which exhibited a higher correlation coefficient than energy expenditure (β = 0.53). The variables accounted for 28% and 36% of the variability, respectively. In contrast, in the multiple regression model, no significant predictive value was found with RMSSD, relative power, fat mass and age on oxidation rate.

**Fig. 1:**
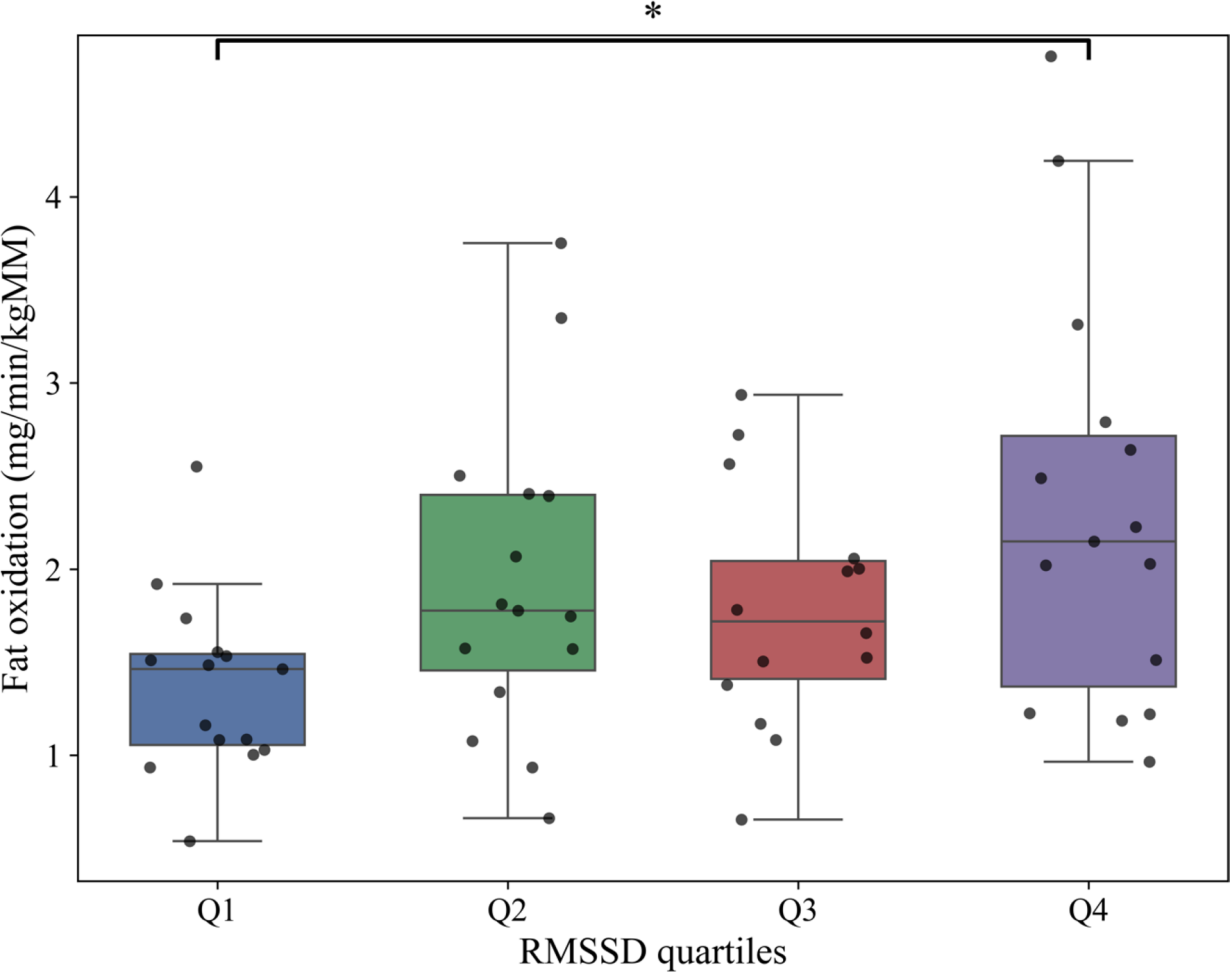
Relative fat oxidation rate across the RMSSD quartiles. *kgMM*, kilograms of muscle mass; *RMSSD*, root mean square of successive beats in miliseconds; cat, category. Q1 ≤ 16.58 ms (n = 16); Q2: > 16.58 − 21.78 ms (n = 15); Q3 ≥ 21.78 − 28.16 ms (n = 14); Q4 > 28.16 ms (n = 15)

For a better understanding, Fig. 1 shows the distribution of the relative rate of fat oxidation (RelFATox) as a function of RMSSD quartiles. An increase in Fat oxidation rate was observed as a function of RMSSD quartiles. In fact, significant differences (*p* = 0.032; F = 3.15) were observed between the lowest RMSSD quartile (Q1; < 16.6 ms) and the highest quartile (Q4; > 28.2 ms). In addition, an increase in the dispersion of the data is evident in the higher quartiles, indicating greater variability in fat oxidation in individuals with higher RMSSD.

**Fig. 2:**
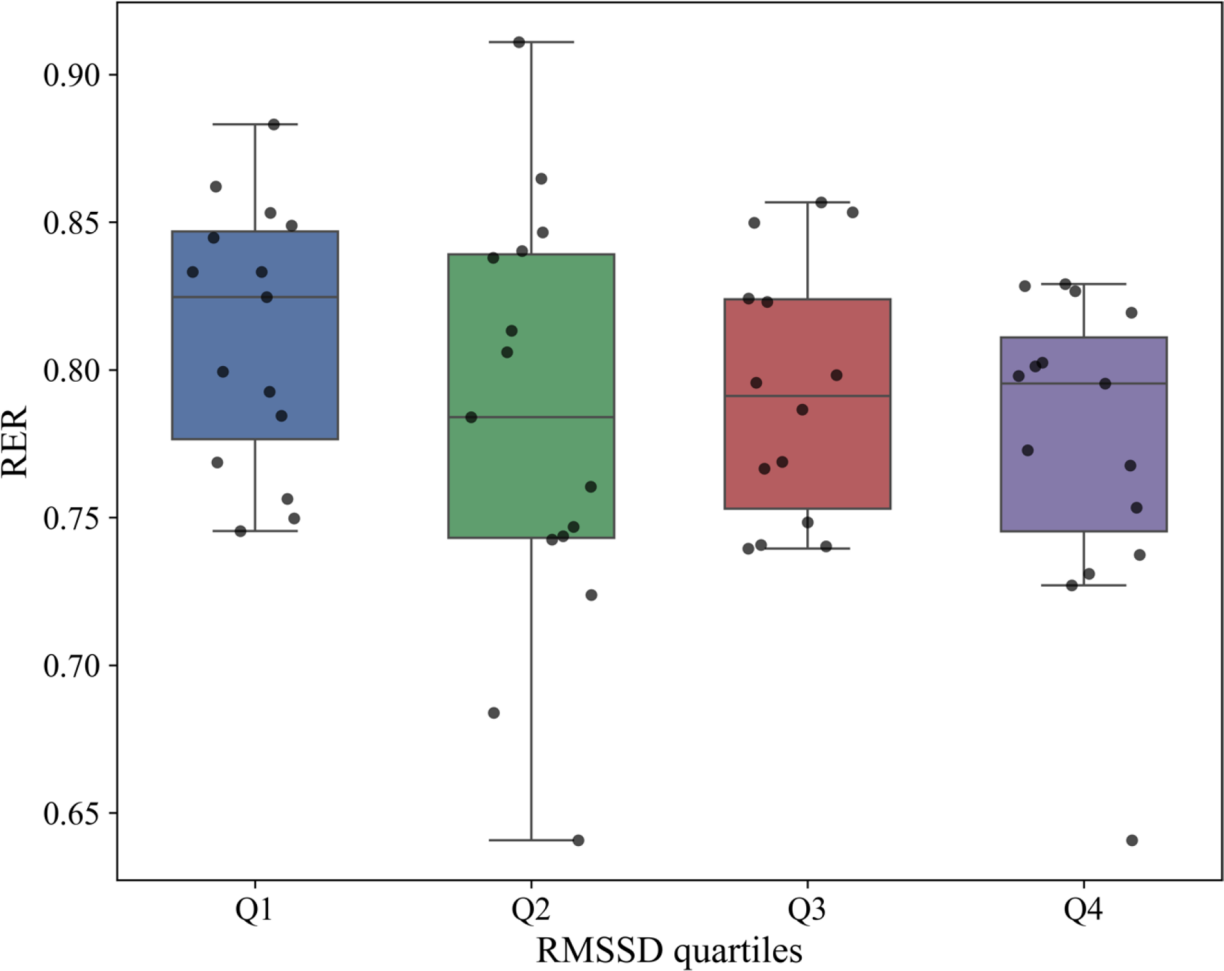
Respiratory exchange ratio across the RMSSD quartiles. *RMSSD*, root mean square of successive beats in milliseconds; cat, category. Q1 ≤ 16.58 ms (n = 16); Q2: > 16.58 − 21.78 ms (n = 15); Q3 ≥ 21.78 − 28.16 ms (n = 14); Q4 > 28.16 ms (n = 15)

Figure 2 shows the distribution of RER as a function of RMSSD quartiles, and in contrast to RelFATox, it does not reflect a trend towards an increase with the RMSSD quartiles, nor show significant differences.

**Fig. 3:**
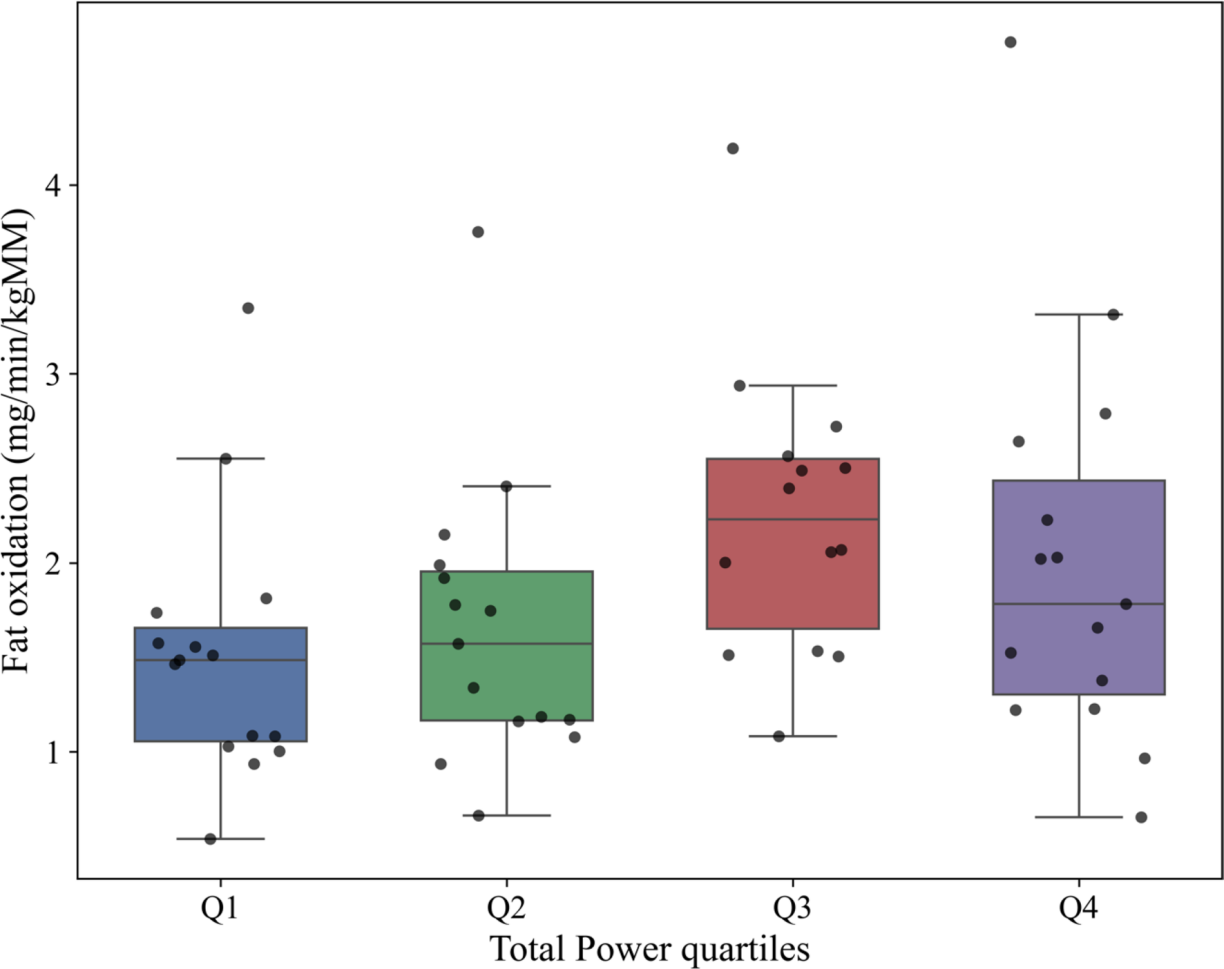
Relative fat oxidation rate across the total power quartiles. *kgMM*, kilograms of muscle mass; Total power, the sum of power across all frequency bands in the power spectral density; cat, category. Q1 ≤ 89.9 ms^2^ (n = 15); Q2: > 89.9 − 340.6 ms^2^ (n = 15); Q3: ≥ 340.6 − 512.5 ms^2^ (n = 15); Q4 ≥ 512.5 ms^2^ (n = 16)

Figure 3 shows the distribution of Fat oxidation rate as a function of Total power quartiles, indicating a similar trend to the RMSSD quartiles until Q3, despite showing no significant differences between Q1 and Q4 (Q1 vs Q3: *p* = 0.08; Q1 vs Q4: *p* = 0.36), due to a large dispersion of the latter.

## Discussion

The main novel finding of this study is the relationship between vagal activity and the rate of Fat oxidation at rest in physically active postmenopausal women. Indeed, the results not only show this association, but also suggest that HRV variable such as Total power of frequency domain, appear to be more influential on FATox than traditionally associated variables as fat mass, muscle power, age, and even energy expenditure under resting conditions.

Postmenopausal women in the late sixties in our sample showed preserved physical fitness as reflected by muscle power above the 75th percentile of the Spanish population [38], and low baseline heart rate [39] and blood lactate levels [40]. In parallel HRV analysis revealed a preserved autonomic behaviour, despite an expected age-associated uniform drop across all parameters, with attenuated sympathetic activation that does not seem to influence basal metabolic regulation [16]. Notably, a remarkable variability in the linear HRV variables traditionally used in autonomic measurements was found. This could be due to the heterogeneous vascular effects of ageing [41], as well as age-related alterations in afferent signalling across integrated cardiovascular and respiratory control pathways [42]. However, this heterogeneity was not reflected in metabolic variables (i.e., FATox), which, despite exhibiting considerable dispersion, showed comparatively less variability than HRV indices, suggesting a relatively more consistent, though not homogeneous, pattern of bioenergetic alteration across participants.

Resting metabolic assessments in these active postmenopausal women reflected a 95% CI of RER (0.78–0.81) still indicating fat oxidation predominance (~ 70%), which exceeded expected values in young healthy adults under similar conditions [14]. It suggests preserved metabolic flexibility at rest [43]. In line with this, the rate of fat oxidation (FATox) was also slightly higher than that observed in younger populations [14], even in the ageing context of lower absolute energy expenditure and BMR [44]. The lower body weight and consequently lower muscle mass [17], may explain the lower BMR. Correlations between BMR and RER were weakly related to each other, yet both were associated with fat oxidation, with stronger correlations with RER. This is expected, as BMR reflects overall resting energy expenditure and includes FATox, whereas RER directly indicates the relative contribution of fat and carbohydrate to energy metabolism.

This suggests, that FATox, could be a more integrative of gas exchange parameters, potentially linked through enhanced oxygen delivery to skeletal muscle through increased cardiac output [45], indicating a close relationship with heart function [46], as well as with autonomic function.

The HRV model confirm the link with FATox, where two important vagal activity biomarkers, RMSSD and Total power, collectively explain up to 30% of the variance of the fat oxidation rate at rest, with higher autonomic function predicting higher resting Fat oxidation rates. It also aligns with recent study supporting the hypothesis that enhanced resting cardiac autonomic control associates with better metabolic flexibility during submaximal exercise [12]. This association likely represents an integrated interaction between autonomic regulation and fat oxidation, often conceptualized as a liver–brain–adipose–neural tissue axis [8], wherein the vagus nerve may modulate fat and carbohydrate mobilization and utilization.

Furthermore, vagal activity counterbalances sympathetic β-adrenergic influences on cardiac function, contributing to the regulation of heart rate and myocardial responsiveness during stress [47]. Additionally, vagal activity has been linked to regulatory pathways affecting mitochondrial redox balance and mitochondrial homeostasis via neuroimmune mechanisms, particularly in the liver and metabolically active peripheral tissues (skeletal muscle and adipose tissue) [48], thus affecting fat oxidation rates [49, 50].

Despite recent findings linking age-related alterations in autonomic nervous system function with changes in ventilatory efficiency and cardiorespiratory integration [51], respiratory frequency (Rf) did not independently explain variability in fat oxidation, notwithstanding exhibiting strong multicollinearity with RMSSD and total power. This pattern is consistent with the notion that respiratory rate captures shared variance within the cardiorespiratory control system, likely mediated by respiratory sinus arrhythmia and central autonomic coupling [52], rather than reflecting an independent determinant of fat oxidation. Breathing frequency is known to covary with vagal-related HRV indices due to shared cardiorespiratory control, without necessarily reflecting metabolic regulation [53]. Under resting or low-intensity conditions, variations in ventilatory pattern are unlikely to constitute a main rate-limiting factors for lipid oxidation. In this context, Rf mainly reflects parallel fluctuations in cardiorespiratory autonomic control, rather than an independent explanatory value for metabolic outcomes.

Regarding the other metabolic outcomes, RER was associated solely with RMSSD, although the categorisation of RMSSD as a function of RER showed no significant trend. The HRV model showed no correlation with BMR, confirming that in this study fat oxidation is the variable most associated with vagal activity in resting conditions.

Also to note, despite the recent association between blood lactate levels and DFA [21], the autonomic nonlinear variable, showed no association with Fat oxidation, RER, or BMR. The complexity of this variable, influenced by multiple signals from electrophysiological, hemodynamic, humoral, and cerebral processes [34, 54], most likely explains the lack of predictive capability to fat oxidation under resting conditions.

The combined predictive model not only highlight the association between two aspects of cardiovascular health, vagal activity and fat oxidation, but also points towards the Total power from the frequency domain outcomes, as a predictive marker of resting Fat oxidation. This variable indicates baroreceptor reflex sensitivity [54]. Notably, as shown by the combined predictive model presented in Table 4, Total power demonstrated an even stronger association with Fat oxidation rate at rest compared to variables such as energy expenditure, which is traditionally closely related to FATox [21]. This observation is particularly significant, as it suggests that the interpretation of the cardiac signals may better predict the Fat oxidation rate rather than total energy expenditure measured simultaneously by the Cosmed K5 metabolic cart. Neither fat mass nor muscular power, despite their previously reported strong association with FATox [55, 56], showed significant correlation with resting Fat oxidation rate in our combined model. Furthermore, RMSSD, strongly associated in the initial model, lost its predictive strength when considering other metabolic variables and ceased to be significantly associated with FATox.

The observed differences between RMSSD and Total power could be due to the different autonomic capacities described for each variable; RMSSD exclusively reflects vagal activity, whereas Total power integrates vagal and other baroreceptor signals [57]. Rather than implying a direct mechanistic pathway, the interaction between baroreflex, respiration, and cardiovascular control has been described as part of a tightly coupled cardiorespiratory regulatory network [58], which may indirectly relate to metabolic processes involved in substrate utilization [59].

As illustrated in Fig. 3, fat oxidation tended to increase from lowest to intermediate Total Power quartiles (Q1-Q3), suggesting an association between greater global autonomic variability and higher fat oxidation. However, this pattern appears to disappear in the highest quartile (Q4), indicating that very high Total Power values (e.g., > 512 ms2) are not necessarily accompanied by proportionally higher FATox. Given the cross-sectional nature of the analysis and the limited sample size, these observations should be interpreted carefully. Longitudinal or interventional studies are needed to determine whether a physiological ceiling or threshold in autonomic variability exists beyond which its association with fat oxidation plateaus in older active women.

RMSSD categorisation is also aligned with this trend across all quartiles, with significant differences in fat oxidation observed between groups with RMSSD values above 28.2 ms compared to those below 16.6 ms. Importantly, this finding highlights RMSSD as a practical marker of vagal modulation that is consistently associated with metabolic variability, while also benefiting from shorter recording durations compared to Total power [10].

Therefore, both RMSSD and Total power appear as predictors of larger rates of fat oxidation in older active postmenopausal women, despite the impaired autonomic function, and mostly lower baroreflex function with age.

Notwithstanding age‐related autonomic changes -arterial stiffening and altered reflex responses, shifts in ANS tone and baroreflex pathways, changes in the sinus node, and a reduced M₂ muscarinic receptor function [60]-, the present findings indicate that variability in vagal-related HRV indices remains meaningfully associated with metabolic outcomes. Moreover, although the increased sympathetic baroreflex response is greater in women due to the loss of the hormones protective effect [60], HRV biomarkers should be viewed as accessible integrative markers of cardiorespiratory regulation rather than direct indicators of specific metabolic mechanisms. This is of particular interest in a physiological comprehensive scenario, where emerging findings reinforce the links between metabolism to breathing and cardiac (autonomic) patterns [61]. From a clinical perspective, this data reinforces the HRV role as a non-invasive tool to monitor autonomic function, particularly the vagus nerve, and identify early dysregulation associated with cardiometabolic risk in aging populations. Clinicians and exercise physiologists may use repeated HRV assessments to guide preventive strategies and for risk stratification. These applications facilitate data-driven decision-making for the prevention and management of age-related cardiometabolic diseases.

Some methodological considerations should be acknowledged. Substrate oxidation was estimated using indirect calorimetry assuming negligible protein oxidation, an approach widely adopted in exercise physiology. While this assumption may introduce a small systematic bias, particularly at rest, it is unlikely to alter the observed associations between autonomic indices and fat oxidation. In addition, despite variables related to ventilation, substrate availability, and peripheral oxygen extraction may also influence metabolic control, the present results consistently indicate that resting autonomic modulation captures meaningful variability in fat oxidation. Future studies incorporating longitudinal designs and complementary physiological measurements may further refine these relationships without detracting from the relevance of the current findings. In addition, further mediation or network-based analytical approaches may help elucidate the relationship between breathing patterns, autonomic modulation, and substrate utilization, thereby offering a more integrative understanding of their interdependence.

### Practical Implications

The insights derived from this study shed light on the interplay between the autonomic nervous system functioning and the rate of Fat oxidation under resting conditions. Specifically, the present findings attribute a potential to RMSSD and Total power as variables associated with resting FATox in these active postmenopausal women. Moreover, the study confirms the influence of the baroreceptor reflex on fat oxidation, attributable to its respiratory modulation. Interestingly, this interaction appears to be confined solely to the rate of Fat oxidation, with no associations with either the respiratory exchange ratio (RER) or basal metabolic rate (BMR) within this cohort of active postmenopausal women. Consequently, these observations suggest that fat oxidation may be a metabolic variable more closely linked to autonomic nervous system activity. Future research should explore the correlation between the baroreceptor reflex and fat oxidation to better understand the mechanisms behind this cross talk, as well as their interaction in other physiological conditions.

## Conclusion

The relationship between baroreflex activity and fat oxidation at rest was found to be significant in active postmenopausal women. Total power emerged as the most significant HRV predictor of its influence on FATox, although RMSSD categorisation also appeared to correlate with the rate of Fat oxidation. The findings of this study advance the understanding of the relationship between autonomic function and fat oxidation, but also propose an alternative approach to explaining resting Fat oxidation by considering HRV variables such as RMSSD and Total power in active postmenopausal women.

## Supplementary Information

Additional file 1 (DOCX 34 KB).

## Data Availability

The datasets generated and/or analyzed during the current study are not publicly available due to the conditions of the ethical approval provided by the Valencia University Human Research Ethics Committee. Notwithstanding, the anonymous data and analysis are available from the corresponding author on reasonable request.

## Abbreviations

ANS: Autonomic nervous system
BMR: Basal metabolic rate
5STS: Five sit to stand test
HRV: Heart rate variability
IPAQ: International physical activity questionnaire
MFO: Maximal fat oxidation
RER: Respiratory exchange ratio
RMSSD: Root mean square of successives differences of heart beats

## Declarations

### Clinical Trial Registration

This study was part of a larger research study investigating associations and changes in Metabolic Flexibility and Autonomic Control After Two Training Programs (Muscle Power vs Metabolic Power), under the acronym POWER Health, registered as a clinical trial (NCT06336070).
