# Supplementary material for: Vagal Activity and Fat Oxidation Basal Correlates in Older Active Postmenopausal Women: A Cross-Sectional Study
Source: Sports Med Open. 2026 Mar 12;12:29. doi: 10.1186/s40798-026-01004-1 (PMC12982731; doi:10.1186/s40798-026-01004-1)
Supplement: Supplementary file 1 — Additional file 1 (DOCX 34 KB). [file 40798_2026_1004_MOESM1_ESM.docx]

**Vagal activity and fat oxidation basal correlates in old active postmenopausal women: a cross-sectional study.**

**Jordi Monferrer-Marín^1^ | Ainoa Roldán^1^ | Jørn Wulff Helge^2^ | Cristina Blasco-Lafarga^1^**

^1^Sport Performance and Physical Fitness Research Group (UIRFIDE), Physical Education and Sports Department, University of Valencia, Valencia, Spain.

^2^Department of Biomedical Sciences, Faculty of Health and Medical Sciences, University of Copenhagen, Copenhagen, Denmark.

**Correspondence:** Cristina Blasco-Lafarga ([m.cristina.blasco@uv.es](mailto:m.cristina.blasco@uv.es))

**Supplementary Materials**

The following supplementary material presents the sequential modeling strategy conducted to identify the optimal set of heart rate variability (HRV) metrics that predict resting metabolic markers, including basal metabolic rate (BMR), respiratory exchange ratio (RER), and fat oxidation (FATox). A total of nine linear regression models were evaluated, each iteratively refined to address statistical assumptions, collinearity issues, and physiological interpretability.

Model 1 included a comprehensive set of HRV variables representing both time, frequency domains, as well as non-linear indices with explained variability of the model of 0.36, that decrease after variables adjustment to 0.24. However, high multicollinearity was observed via variance inflation factors (VIFs), it was therefore necessary to reduce the number of predictors.

# **Model 1**

| **Variable** | **Estimate** | **Std. Error** | **t value** | **Pr(>\|t\|)** |
| --- | --- | --- | --- | --- |
| **Intercept** | -0.67 | 1.61 | 0.56 | 0.58 |
| **PNS index** | -0.23 | 0.44 | -0.34 | 0.73 |
| **SNSindex** | -0.03 | 0.21 | -0.09 | 0.92 |
| **RMSSD** | 0.05 | 4.43 | 0.22 | 0.83 |
| **LF power** | 0.00 | 0.00 | 0.17 | 0.87 |
| **HF power** | 0.00 | 0.00 | 0.17 | 0.86 |
| **TotalPower** | -0.00 | 0.00 | -0.08 | 0.93 |
| **SD1** | -0.03 | 6.25 | -0.21 | 0.83 |
| **DFA** | 0.42 | 0.84 | 0.13 | 0.90 |
| **SampEn** | 0.71 | 0.66 | 0.11 | 0.91 |

Several HRV-derived variables showed severe multicollinearity (SD1: VIF = 1.93 × 10⁶; RMSSD: VIF = 1.93 × 10⁶; LF power: VIF = 163.9; HF power: VIF = 292.1; total power: VIF = 738.2; PNS index: VIF = 31.4). To address this issue and ensure a parsimonious and interpretable model, SD1, LF power, and the PNS index were excluded for Model 2 due to their redundancy with RMSSD and total power, consistent with the well-documented overlap among parasympathetic-related HRV indices, increasing the adjusted R² to 0.28.

# **Model 2**

| **Variable** | **Estimate** | **Std. Error** | **t value** | **Pr(>\|t\|)** |
| --- | --- | --- | --- | --- |
| **Intercept** | -0.80 | 1.56 | 0.52 | 0.61 |
| **SNSindex** | 0.05 | 0.10 | 0.50 | 0.62 |
| **RMSSD** | 0.01 | 0.00 | 2.07 | 0.04 |
| **HF power** | 0.00 | 0.00 | -0.04 | 0.97 |
| **TotalPower** | 0.00 | 0.00 | 0.77 | 0.44 |
| **DFA** | 0.16 | 0.71 | 0.23 | 0.82 |
| **SampEn** | 0.11 | 0.62 | 0.18 | 0.86 |

Model 3 further refined the specification by removing HF power. Although HF power is traditionally considered a marker of vagal modulation, it showed substantial multicollinearity (VIF = 12.53), largely overlapping with RMSSD (VIF = 5.16) and total power (VIF = 12.44), without providing additional explanatory value. Its exclusion reduced collinearity while preserving the physiological interpretability and parsimony of the model. The resulting model yielded significant associations between RMSSD and Total Power with FATox, suggesting their stronger predictive utility. Specifically, this model explained 37.3% of the variance in FATox (adjusted R² = 31.4%) and reached statistical significance (F = 6.302, p < 0.001). However, the large number of multi-domain variables, and the null association with SNS index, necessitated further refinement of the model.

# **Model 3**

| **Variable** | **Estimate** | **Std. Error** | **t value** | **Pr(>\|t\|)** |
| --- | --- | --- | --- | --- |
| **Intercept** | -0.626786 | 1.5723746 | -0.399 | 0.69177 |
| **SNSindex** | 0.0736279 | 0.0976272 | 0.754 | 0.45408 |
| **RMSSD** | 0.0191296 | 0.006841 | 2.796 | 0.00719 |
| **TotalPower** | 0.0002861 | 0.0001418 | 2.017 | 0.04874 |
| **DFA** | 0.4891026 | 0.6118371 | 0.799 | 0.42763 |
| **SampEn** | 0.6755797 | 0.5861581 | 1.153 | 0.25426 |

Model 4 represents the final HRV-only model and the first to be included in the main manuscript (Table 1). This model retains one variable per HRV domain (RMSSD for time, Total Power for frequency, DFA for non-linear), with SNS and SampEn excluded due to statistical insignificance and overlapping explanatory variance. This parsimonious configuration achieved robust model fit, reaching an adjusted R² of 31.9%, with a total R² of 35.4% and F = 10.06 (p < 0.001).

# **Model 4**

| **Variable** | **Estimate** | **Std. Error** | **t value** | **Pr(>\|t\|)** |
| --- | --- | --- | --- | --- |
| **Intercept** | 1.1071227 | 0.5779022 | 1.916 | 0.0606 |
| **RMSSD** | 0.01268 | 0.0043352 | 2.925 | 0.005 |
| **TotalPower** | 0.0002574 | 0.0001236 | 2.082 | 0.042 |
| **DFA** | 0.1753515 | 0.5248274 | 0.334 | 0.7396 |

To assess the specificity of HRV variables in predicting FATox, Models 5 and 6 replicated Model 4 using RER and BMR as dependent variables, respectively. These models revealed markedly lower explained variance in RER, with values of 17.0% of variance (Adjusted R² = 12.6%), with an F-statistic of 3.826 (p = 0.0145). This suggested that RER is less tightly coupled to autonomic markers than FATox.

# **Model 5**

| **Variable** | **Estimate** | **Std. Error** | **t value** | **Pr(>\|t\|)** |
| --- | --- | --- | --- | --- |
| **Intercept** | 0.8474 | 0.04161 | 20.364 | <2e-16 |
| **RMSSD** | -0.0009693 | 0.0003122 | -3.105 | 0.00298 |
| **TotalPower** | 8.285e-06 | 8.9e-06 | 0.931 | 0.35592 |
| **DFA** | -0.03705 | 0.03777 | -0.981 | 0.33082 |

Targeting BMR instead of FATox, Model 6’s explanatory power dropped further (R² = 6.1%, Adjusted R² = 1.0%) with F = 1.203 and p = 0.317, indicating no significant association between HRV variables and resting metabolic rate. Models 5 and 6 reinforce the hypothesis that HRV indices, particularly RMSSD and Total Power, are more tightly coupled with FATox than with whole-body energy expenditure or substrate partitioning at rest.

# **Model 6**

| **Variable** | **Estimate** | **Std. Error** | **t value** | **Pr(>\|t\|)** |
| --- | --- | --- | --- | --- |
| **Intercept** | 1059.24949 | 217.05625 | 4.88 | 9.19e-06 |
| **RMSSD** | 1.07823 | 1.6283 | 0.662 | 0.511 |
| **TotalPower** | 0.01382 | 0.04642 | 0.298 | 0.767 |
| **DFA** | -142.53475 | 197.00674 | -0.724 | 0.472 |

Model 7 shows the multivariable linear regression model including respiratory frequency (Rf) together with heart rate variability (HRV) indices (RMSSD, total power, and DFA). In this model, none of the predictors reached statistical significance, including RMSSD, total power, DFA, or Rf, indicating the absence of an independent association of these autonomic variables with the dependent outcome when considered simultaneously. Importantly, multicollinearity diagnostics revealed a very high variance inflation factor for RMSSD (VIF > 20), suggesting substantial redundancy between RMSSD and other HRV-related predictors in the model.

# **Model 7**

| **Variable** | **Estimate** | **Std. Error** | **t value** | **Pr(>\|t\|)** |
| --- | --- | --- | --- | --- |
| **Intercept** | 1.0631 | 1.4506 | 0.733 | 0.468 |
| **RMSSD** | 0.0089 | 0.0337 | 0.265 | 0.793 |
| **Rf** | 0.0157 | 0.0390 | 0.403 | 0.689 |
| **TotalPower** | 0.0003 | 0.0005 | 0.537 | 0.595 |
| **DFA** | 0.0680 | 1.0112 | 0.067 | 0.947 |

Given this marked multicollinearity, a second model excluding RMSSD was computed (Table 8) to improve model stability and interpretability. In this reduced model, the explained variance of the model was 25%, with total power emerging as a significant predictor, while DFA and Rf remained non-significant. These findings indicate that the inclusion of RMSSD in the full model masked the independent contribution of total power due to shared variance among HRV indices with Rf presence. Therefore, Table 8 is presented as a complementary model that mitigates multicollinearity effects and more clearly isolates the predictive role of global autonomic power, while respiratory frequency does not independently explain additional variance in either model.

# **Model 8**

| **Variable** | **Estimate** | **Std. Error** | **t value** | **Pr(>\|t\|)** |
| --- | --- | --- | --- | --- |
| **Intercept** | 1.3577 | 0.9197 | 1.476 | 0.148 |
| **Rf** | 0.0167 | 0.0384 | 0.436 | 0.665 |
| **TotalPower** | 0.0004 | 0.0001 | 3.295 | 0.002 |
| **DFA** | -0.1414 | 0.6226 | -0.227 | 0.821 |

Similarly, a complementary model excluding total power (Table 9) was calculated to further address multicollinearity among HRV indices. In this reduced specification, the model explained a comparable proportion of variance (R² = 25%), with RMSSD as the only significant predictor, showing a slightly stronger standardised effect (β = 0.55), while DFA and respiratory rate remained non-significant. These results mirror those observed in model 8 and indicate that RMSSD and total power capture autonomic information that may overlap. This occurs when combined with respiratory rate, obscuring their individual contributions; however, when considered separately, each metric independently explains a similar proportion of the variance. Consequently, Table 9 is presented as a complementary model that isolates the predictive role of vagus nerve-mediated autonomic modulation, confirming that respiratory rate does not independently contribute additional explanatory power in either specification, and even reduces the predictive power of the model (25% of variance explained) compared to model 4 with HRV variables alone (30%), given the similarities with RMSSD and Total Power.

# **Model 9**

| **Variable** | **Estimate** | **Std. Error** | **t value** | **Pr(>\|t\|)** |
| --- | --- | --- | --- | --- |
| **Intercept** | 0.5466 | 1.0759 | 0.508 | 0.614 |
| **RMSSD** | 0.0264 | 0.0081 | 3.252 | 0.002 |
| **DFA** | 0.4471 | 0.7172 | 0.623 | 0.536 |
| **Rf** | 0.0125 | 0.0382 | 0.328 | 0.744 |

Model 10 sought to contextualize HRV predictors, without Rf given its low influence on RelFATox, by introducing fat mass and relative power output in a modified version of Model 4. Interestingly, RMSSD remained a significant predictor, and model performance surpassed 30% explained variance, suggesting a physiologically plausible link between vagal tone and lipid metabolism efficiency.

# **Model 10**

| **Variable** | **Estimate** | **Std. Error** | **t value** | **Pr(>\|t\|)** |
| --- | --- | --- | --- | --- |
| **Intercept** | 1.612481 | 1.091184 | 1.478 | 0.14731 |
| **RMSSD** | 0.022321 | 0.006222 | 3.587 | 0.000901 |
| **FM_PRE** | 0.012441 | 0.018407 | 0.676 | 0.502979 |
| **RelPower** | -0.220012 | 0.211091 | -1.042 | 0.303548 |

# Model 11 substituted RMSSD for Total Power with no noticeable change, reaching 31.0% explained variance and 25.8% adjusted variance (F = 5.992, p = 0.0018), which is why none of the HRV variables were removed and were combined in a final model, along with the age and energy expenditure variables, to combine the HRV variables with those traditionally associated with FATox.

# **Model 11**

| **Variable** | **Estimate** | **Std. Error** | **t value** | **Pr(>\|t\|)** |
| --- | --- | --- | --- | --- |
| **Intercept** | 2.0077515 | 0.9905388 | 2.027 | 0.049049 |
| **TotalPower** | 0.0004358 | 0.0001097 | 3.974 | 0.000272 |
| **FM_PRE** | 0.0119585 | 0.0173227 | 0.69 | 0.49378 |
| **RelPower** | -0.275489 | 0.1938136 | -1.421 | 0.16258 |

Finally, Model 12 integrated RMSSD and Total Power along with fat mass and relative power output, age and energy expenditure, reflect significantly improved performance, explaining 57.5% of the variance in FATox (Adjusted R² = 50.4%). It yielded an F-statistic of 8.116 with p < 0.001. This model captured complementary contributions from autonomic function and body composition and was therefore selected as the second key model in the main manuscript.

# **Model 12**

| **Predictor** | **Estimate** | **Std. Error** | **t value** | | **Pr(>\|t\|)** |
| --- | --- | --- | --- | --- | --- |
| **Intercept** | 0.77704 | 1.86358 | 0.417 | | 0.679 |
| **RMSSD** | -0.02245 | 0.02029 | -1.107 | | 0.276 |
| **Total Power** | 0.00076 | 0.00037 | 2.037 | | 0.049 |
| **Fat Mass** | -0.00140 | 0.01612 | | -0.087 | 0.931 |
| **Relative 5STS Power** | -0.27114 | 0.19815 | -1.368 | | 0.180 |
| **Energy Expenditure** | 1.89935 | 0.42776 | 4.440 | | <0.001 |
| **Age** | 0.00260 | 0.01938 | 0.134 | | 0.894 |

The modelling process not only followed rigorous statistical refinement (e.g., stepwise exclusion based on p-values and VIFs), but also reflected theoretical considerations. Specifically, the models were constrained to retain one HRV variable per physiological domain to avoid redundancy, maximize interpretability, and promote translational applicability. This strategy supports a mechanistic interpretation of how different components of autonomic regulation (vagal tone, overall autonomic output, and signal complexity) may differentially relate to metabolic outcomes.
